# The Influence of Different Social Roles Activation on Women’s Financial and Consumer Choices

**DOI:** 10.3389/fpsyg.2016.00365

**Published:** 2016-03-17

**Authors:** Katarzyna Sekścińska, Agata Trzcińska, Dominika A. Maison

**Affiliations:** Faculty of Psychology, University of WarsawWarsaw, Poland

**Keywords:** women’s social roles, traditional vs. non-traditional social role, financial choices, consumer choices, product judgment

## Abstract

In recent times, the changes occurring in the social role of women and men have been observed. Traditionally, the dominating social role of the woman was as housewife, and that of the man was focused on work and family maintenance. Nowadays, the social role of women is evolving in the direction of taking a profession, while increasingly men are taking care of the household. The main goal of the studies presented here was to verify how the activation of different social roles (traditional or non-traditional) may be reflected in women’s financial and consumer choices. Three experimental studies were conducted. In the first study (*n* = 195 females), three different social roles of women – professional (non-traditional), housewife (traditional) and neutral (control) – were activated. The results showed that activating women’s non-traditional social role increased their tendency to invest and decreased their propensity to save money compared to the activation of the traditional or neutral social role. The goal of the second study (*n* = 196 females) was to check whether, despite there being no differences in the level of consumption in the first study, can any differences be observed in the preference for the type of products chosen for consumption. The results showed that activating the non-traditional social role raised the propensity to spend funds on products and services for individual use and reduced the willingness to buy goods for collective use (shared with other members of the household). The purpose of the third study (*n* = 90 females) was to examine how different images of women appearing in advertisements may affect women’s judgments of the advertised product. Women who watched the ad with woman in the non-traditional social role estimated the product quality, look, color and price higher that participants exposed to the advertisement presenting the woman in traditional or neutral social role. The present studies give some evidence that the new, non-traditional social role of women that is often presented in the media may affect women’s everyday financial choices and judgments of products.

## Introduction

The traditional social roles of women and men have remained unchanged for many years (Barska, 2005; Diekman and Goodfriend, 2006). The traditional social role of women is that of the lady of the house, taking care of the family, being focused on children and their happiness. Traditional occupational roles attributed to women are related to caring for others (e.g., homekeeper, nurse) and require communal characteristics (e.g., kind, sensitive) (Eagly et al., 2000). On the other hand, the traditional man is the “head” of the family, the one who is responsible for the maintenance of the house and who, through strength and determination, defends it against dangers. Traditionally men occupy the roles related to leadership (e.g., manager), which are associated with agentic characteristics (e.g., independent, competitive) (*ibidem*). The traditional gender social roles are consistent with stereotypical traits attributed to men and women. In most countries, attributes such as affection or sensitivity are considered more typical of women, whereas attributes such as aggressiveness or courage are considered more typical of men (see Williams and Best, 1990; Williams et al., 1999). However, in the last two decades, along with the deepening social and economic changes, the social roles of women and men began to undergo vivid modifications.

Since the mid-20th century an increasing number of women can be observed in the labor market. Moreover, women have entered into male-dominated roles, e.g., leadership roles, including in politics. Since 1965, the proportion of women who have majored in business, medicine and law has risen significantly (Astin et al., 2002). The modern world has created a new role for women, which is gradually approaching that of the traditional social role of men (e.g., working, earning money). At the same time, men’s work roles have remained relatively stable and men continue to participate full-time in the paid labor force (Fullerton, 1999; England, 2006). However, today, the role of the man is becoming slightly closer to the traditional role of women (e.g., taking care of the family) (Arcimowicz, 2008; Bajkowski, 2010), although men are generally perceived as maintaining higher levels of male- rather than female-stereotypic characteristics (Diekman and Goodfriend, 2006). Taking into account the social role theory (Diekman and Eagly, 2000), which assumes that the behavior of group members shapes their stereotype, it is worth examining what are the consequences of such gendered social change.

The trend of gendered social change is reflected in the media: television, newspapers, and advertisements (Bator, 1998; Arcimowicz, 2003; Maison and Rudzińska, 2009; Mager and Helgeson, 2011), which may facilitate further social change. A recent review of 50 years of US magazine advertising (Mager and Helgeson, 2011) indicated that advertisements no longer show that a woman’s place is only at home, as stated by Courtney and Lockeretz (1971). According to Bator (1998), we can distinguish two main images of women created by the modern world: the traditional woman, the lady of the house (the traditional role), and a non-traditional woman – who is focused on herself, confident, working, professional, often a businesswoman. As can be seen, in the modern world, in addition to the traditional social roles of women, a new role has been created: a woman approaching the traditional social role of a man. Changes in the social roles of women and men are connected largely with the change of priorities and focus (me vs. others). For women the change is taking them in the direction of a stronger concentration on themselves and their work (individualism), while for men it is leading to a more social direction – concentration on home, family and children (Eagly and Diekman, 2003), although, as mentioned earlier, the change in gender social roles has been less extensive for men than women (*ibidem*). New roles of women and men have emerged and have been adopted by the media; however, there is only a little research investigating how and to what extent activation of those new social roles may affect people’s judgments and behaviors in various areas.

One of the few studies examining how individuals adapt to social change was conducted by Diekman et al. (2013). The study showed that people accommodate self-relevant cognitions and behaviors to societal change. Participants who learned about social change for their gender in-group expected greater personal success in gender-based non-traditional careers. Moreover, after learning about women entering non-traditional roles, participants more often sought out information about leadership (non-traditional choice) than information about physical appearance (traditional choice), which means that learning about social change in the roles of women had some behavioral consequences. The above-mentioned study provided the evidence that exposure to social change can influence people’s self-relevant cognitions and behaviors in a way that facilitates further social change. If so, it is possible that exposure to different images of gender roles (traditional vs. non-traditional) could change people’s judgments and behaviors.

In our study we investigated whether the activation of the traditional or non-traditional social role of women can influence women’s choices. We decided to study only women, because changes in the social roles of women are definitely more extensive than those of men (Eagly and Diekman, 2003) and the consequences of changes in women’s social role may be more important for societal change. In particular, we investigated the influence of the activation of traditional vs. non-traditional women’s social roles on female financial choices, where the non-traditional role was similar to the traditional social role of men. Differences between women and men in financial decisions and behaviors have been repeatedly proven (e.g., Bajtelsmit and Bernasek, 1996; Maison, 2013). Nevertheless, it is worth asking if those differences will remain unchanged in a world where gender social roles evolve.

To date, researchers report that women are, in general, more risk averse (Bajtelsmit and Bernasek, 1996; Bajtelsmit and VanderHei, 1997; Bajtelsmit et al., 1999; Grable, 2000; Hallahan et al., 2004; Faff et al., 2008; Neelakantan, 2010); this has also been confirmed in a meta-analysis of over 150 studies conducted by Byrnes et al. (1999). The results pointed to a greater overall propensity of men than women to take various types of risk (e.g., the use of drugs, smoking cigarettes, drinking alcohol, risky sexual behaviors, the way of driving the car and many others). Following the recommendation of Barke et al. (1997), who stated that explanations of gender differences in risk aversion should be sought by examining social roles, we wondered whether changes in social roles may be reflected in the modification of people’s financial behaviors.

It was also confirmed that men are more prone to taking financial risks than women, manifesting in a tendency to gamble, picking aggressive financial instruments and building investment portfolios with a high level of risk (Byrnes et al., 1999). A number of studies showed that women, due to their higher risk aversion, are more conservative in their investment decisions than men (Bajtelsmit and Bernasek, 1996; Hinz et al., 1997; Bajtelsmit et al., 1999). These studies revealed that women hold a much higher proportion of their portfolios in fixed assets and are less likely to invest in employer stock and equities than men. Taking those results into account, we wondered whether changes in social roles may modify the investment behaviors of an individual.

The basic financial behavior that can reduce financial risk is saving. This provides resources for retirement, enables financing unexpected losses of income and allows a more stable consumption to be maintained over time (consumption smoothing). In the literature some evidence can be found of gender differences in saving behavior (Hungerford, 1999; Seguino and Floro, 2003) and the amount of savings (Fawcett Society, 2007). In a study of 20 semi-industrialized countries, Seguino and Floro (2003) found that women are more likely than men to save. However, findings from the United Kingdom indicate that although women have the same propensity as men to save, they tend to accumulate fewer saving than men (Fawcett Society, 2007). One of the explanations for this result is the fact that women generally put family consumption above personal consumption (Fawcett Society, 2007). Seguino and Floro (2003) stated that the factors that affect women’s and men’s propensity to save may be contradictory in their effect. Women reveal more care responsibility for the household, which may lead to more consumption spending and thus less saving (*ibidem*). On the other hand, this responsibility may lead women to save more than men for precautionary reasons, due to their stronger need to smooth family consumption. Differences between men and women in their saving behaviors may suggest that the salience of different social roles of women (traditional vs. non-traditional) may lead women to different saving choices. The non-traditional role could elicit thinking in a rather masculine way, while the traditional role activation could elicit thinking in a feminine way.

Other gender differences in financial decisions and behaviors concern consumption choices. According to Czuchaj-Ładód and Stopyra (2015) polish women do everyday shopping more often than men, buying clothes, medicines, cosmetics, items for the home and products for children. On the other hand, men buy electronics, cars and alcohol more often. They also like to spend money on sport and entertainment. The above-mentioned results indicate that while women exhibit communal behavior (e.g., everyday shopping, items for home, products for children) in their shopping, men are more focused on their own needs (e.g., electronics, sport entertainment). The source of those differences could be a higher tendency to take care of the household by women than men, which is in accordance with the stereotypical social role of women (Seguino and Floro, 2003). The creation of the new role of women (very close to the early stereotype of the social role of men) probably changes women’s judgments and choices regarding consumption. In our study we will investigate whether the activation of different social roles of women can influence consumer choices.

The findings from the previous studies showing different financial behavior of women and men (Bajtelsmit and Bernasek, 1996; Maison, 2013) prompt the question of whether this difference is an immanent and stable feature of women and men or whether it can be modified by external influences. The main question of the studies presented here was whether the activation of the traditional or non-traditional social role of women can influence their financial choices (consumption, saving and investing). So far, no scientific research has provided the answer to this question. We conducted three experimental studies where we hypothesized that the thinking of different social role activation affects women’s decisions related to consumption, saving and investing.

We expected that exposure to the non-traditional social role would make women more prone to invest their money in assets associated with some level of risk, while the activation of the traditional role would increase participants’ tendency to save money in assets that are not risky (Study 1). Study 2 aimed at examining the impact of the activation of different social roles on consumer choices. Because the new social role of women is connected with a stronger concentration on themselves and their work (and not only on home and family), we expected that the activation of the non-traditional social role would make women more prone to spend their money on products for individual use (e.g., clothes, jewelry, shoes, color cosmetics) and services related to relaxation (e.g., holidays, spa). On the other hand, we expected that after activation of the traditional social role, women’s tendency to spend money on functional consumption like food and hygiene products or equipment for the family (e.g., furniture, TV set, household goods) would increase. The goal of the last study was to verify how the different social roles of women presented in advertisements may change women’s judgments of the advertised product and the willingness to buy it.

## Study 1 – the Influence of Women’s Social Roles on their Financial Decisions

The main goal of this study was to examine whether and how the activation of different social roles of women may affect their propensity to consume, save and invest. The starting point for deliberations on consumption and saving is the consumption function presented by Keynes (1936/2014). The amount that households spend on consumption depends “*partly on the amount of its income, partly on other objective attendant circumstances, and partly on the subjective needs and the psychological propensities and habits of the individuals composing it and the principles on which the income is divided between them (ibidem.* Chapt. 8).” Keynes concluded that real disposable income is the most important determinant of consumption and saving. He put forward a psychological law of consumption, according to which, as income increases consumption increases but not by as much as the increase in income (*ibidem*). In other words, marginal propensity to consume is more than zero but less than one. In Keynes’s theory the average propensity to consume (APC) decreases with increasing income (*ibidem*). Thus the greater the disposable income, the higher the part of the income allocated for savings should be. Early empirical findings supported Keynes’s conjectures about consumption (e.g., Hall and Taylor, 1993). But in the 1940s, empirical studies of long-term time series data from the US economy for the period 1869–1938 by Simon Kuznets showed that consumption was in fact stable in spite of rising income, and APC was relatively stable over long periods. Further studies confirmed Kuznets’s findings (e.g., Snowdown and Vane, 2005). Evidence therefore indicated that there are two different consumption functions: a short-term consumption function with the variable value of the APC, and a long-run consumption function in which the APC was constant regardless of the level of income. A short-term consumption function seemed to confirm Keynes’s conjectures. According to Keynes’s theory, in the short-term people manage a little amount of disposable money differently from a large amount (Samuelson and Nordhaus, 1998), therefore in the current studies the financial choices were tested in three situations, where each person has a small, medium and large amount of disposable money.

### Method

#### Participants

In the first study 195 women participated, students of the fourth and fifth years at University of Warsaw, aged 22–32 years (*M* = 24, *SD* = 1.91). The Ethics Board of the Faculty of Psychology at the University of Warsaw approved the study, which was carried out in accordance with the Board’s recommendations. At the end of the study, the participants were fully debriefed.

#### Experimental Design and Materials

The study was conducted with three conditions: two experimental conditions and one control group. Participants were randomly assigned to each condition where the traditional, non-traditional or neutral social role of women was activated. The social role that was activated was the between subjects IV. The first within subjects IV was the financial activity measured on three levels: consumption, saving, investing. The second within subjects IV was the level of the amount that participants were asked to distribute, measured on three levels: small (PLN 500; ∼USD 135), medium (PLN 3000; ∼USD 800) and large (PLN 10,000; ∼USD 2655). Women’s propensity for different financial choices indicated by the amount of money assigned by the participants to different categories was the DV.

Different social roles were activated using photos of a woman who was standing next to a table with chairs in a bright room with a cup of tea in her hand. There were three versions of the picture in which the woman’s outfit differed. One photo showed a woman in everyday clothes with a kitchen towel and oven glove (traditional social role). The second version of the picture showed person in the role of a modern woman, inconsistent with the traditional stereotype (non-traditional social role). She was in clothes suggesting her professional position. In the third version, the woman on the picture was neutral in terms of social role, wearing clothes not associated with any social role (neutral/control). The effectiveness of manipulation was checked in the pilot study. Participants of the pilot study were shown the set of pictures and after viewing each picture they were asked to answer the question “*who was the woman in the picture?*” Final versions of pictures were chosen based on the characteristics that were assigned to each picture. The woman on the final version of the traditional picture was described, among others, as housewife, lady of the house, mother taking care of her children, woman focused on house and family. The woman in the picture representing the non-traditional social role was described, among others, as earning for herself, financially independent, a businesswoman, worker, higher level employee, working in an important position. The woman in the control picture was described, among others, as smiling, nice, cheerful person, girl next door or student. There were no descriptions related to the occupational status.

The propensity for different financial choices indicated by the amount of money assigned by participants to different categories was measured using the tool in which participants were asked to distribute a small (PLN 500; ∼USD 135), medium (PLN 3000; ∼USD 800) and large (PLN 10,000; ∼USD 2655) amount of money between consumption, saving and investing. The values of the small, medium and large amounts were established on the basis of a pilot study. The tool also included the information of what consuming, saving, and investing meant in the context of the study: Consuming meant spending money on products or services; Saving meant keeping the money in non-profitable (or almost non-profitable) form, without the risk of loss, e.g., deposit in a non-interest-bearing bank account. Investing was defined as allocating funds to financial instruments that can generate profits but with the risk of losses, e.g., stocks or mutual funds.

#### Procedure

At the beginning of the study students were informed that the researcher was conducting two independent, short pilot studies. The researcher asked them if they were happy to take part in both of them. Participants were asked to take a look at one picture of woman carefully for 30 s (the researcher controlled the time). This was either a picture of a woman in the traditional social role (I experimental condition), or of a woman in the non-traditional social role (II experimental condition) or a picture of a woman who was neutral in terms of social role (control group). That was an experimental manipulation. After viewing the picture, participants were asked to write down the answers to two questions: “*Who was the woman in the viewed picture?*” and “*Would she be a good actress for an advertisement in the press?*”. The questions had two aims: (1) To make participants sure that there was a reason to look at the picture; and (2) To check the experimental manipulation (question 1). After that, participants were informed that it was the end of the first study and they were asked to take part in another one. The researcher informed participants that the next study was about financial choices. Then the participants’ propensity for different financial choices (consumption, saving, investing) was measured. At the end the participants were fully debriefed.

### Results

A 3 (social role) × 3 (financial activity) × 3 (amount level) mixed-design analysis of variance (ANOVA) was conducted. A significant main effect of social role was observed (*F*[2,191] = 3.23, *p* < 0.05, η^2^ = 0.03, **Table 1**). A significant difference in the amount of money assigned between non-traditional and traditional groups occurred (*p* < 0.05). There was no significant difference between the control and the traditional or non-traditional groups. The second main effect (of financial activity) was significant (*F*[1.57,299.75] = 25.03, *p* < 0.001, η^2^ = 0.12, **Table 1**). There were significant differences in the amount of money assigned between consumption and both saving (*p* < 0.001) and investing (*p* < 0.001). There was no significant difference between saving and investing. The third main effect (amount level) was also significant (*F*[1.52,291.10] = 9153.86, *p* < 0.001, η^2^ = 0.98, **Table 1**). The differences between small and both medium (*p* < 0.001) and large (*p* < 0.001) amount of money assigned were significant. Moreover, the difference between medium and large amount level was also significant (*p* < 0.001).

**Table 1:** Mean amounts of money (in PLN) assigned depending on social role, financial activity and amount level.

	*M*	95% Confidence Interval
Social role
Non-traditional	1512.56	[1477.02, 1548.11]
Traditional	1448.38	[1412.83, 1483.92]
Control	1487.76	[1451.94, 1523.58]
Financial activity
Consumption	937.70	[831.43, 1043.92]
Saving	1719.64	[1554.42, 1884.87]
Investing	1791.35	[1611.39, 1971.32]
Amount level
Small	165.89	[163.80, 167.99]
Medium	1003.97	[971.76, 1036.18]
Large	3278.84	[3229.51, 3328.17]

The two-way interaction between social role and financial activity was significant (*F*[3.14,299.75] = 7.63, *p* < 0.001, η^2^ = 0.07). However, the two-way interaction between social role and amount level was not significant (*F*[3.04,291.10] = 1.09, *p* = 0.35, η^2^ = 0.01). The interaction between financial activity and amount level was significant (*F*[1.63,311.97] = 35.87, *p* < 0.001, η^2^ = 0.16).

Moreover, the three-way interaction between social role, financial activity and amount level was also significant (*F*[3.27,311.97] = 7.10, *p* < 0.001, η^2^ = 0.07).

In order to perform the follow-up tests, the data set was split according to the levels of the variables involved in the significant three-way interaction. In a first step, the data set was divided into the three amount levels (small, medium, large). Three mixed-design 3 (social roles) × 3 (financial activity) ANOVAs were conducted, each for a different level of amount. For the small amount the main effect of social role was not observed (*F*[2,192] = 0.08, *p* = 0.93, η^2^ = 0.001, **Table 2**). The main effect of spending was significant (*F*[2,384] = 38.15, *p <* 0.001, η^2^ = 0.17). There were significant differences observed in money assigned between consumption and both saving (*p* < 0.001) and investing (*p* < 0.001) and also between saving and investing (*p* < 0.001). The interaction between social role and financial activity was not significant for the small amount level (*F*[4,384] = 0.95, *p* = 0.44, η^2^ = 0.01).

**Table 2:** Mean amounts of money (in PLN) assigned depending on social role, financial activity for small, medium and large amount level.

	Small amount *M*; 95% CI	Medium amount *M*; 95% CI	Large amount *M*; 95% CI
Social role
Non-traditional	166.41; [162.80, 170.02]	1049.23; [993.73,1104.73]	3322.05; [3236.83, 3407.27]
Traditional	165.39; [161.78, 168.99]	939.23; [883.73, 994.73]	3240.51; [3155.29, 3325.74]
Control	165.90; [162.29, 169.51]	1023.08; [967.58, 1078.58]	3273.96; [3188.07, 3359.84]
Financial activity
Consumption	236.28; [217.04, 255.52]	779.74; [704.21, 895.28]	1776.86; [1535.52, 2018.17]
Saving	166.15; [147.50, 184.81]	1180.00; [1045.62, 1314.38]	3811.62; [3407.25, 4215.99]
Investing	95.26; [77.67, 112.84]	1031.80; [905.22, 1158.37]	4248.05; [3807.91, 4688.18]

For the medium level of money the main effect of social role on money was observed (*F*[2,192] = 4.17, *p <* 0.05, η^2^ = 0.04, **Table 2**). Significant differences in money assigned were observed between the non-traditional and traditional groups (*p* < 0.05). No difference was observed between the control and both traditional and non-traditional groups. A significant main effect of financial activity also occurred (*F*[1.85,354.86] = 7.12, *p* = 0.001, η^2^ = 0.04, **Table 2**). The significant differences in money assigned were observed between consumption and both saving (*p* < 0.001) and investing (*p* < 0.05). There was no significant difference between saving and investing. Furthermore, the interaction between social role and financial activity was significant (*F*[3.70,354.86] = 4.33, *p* < 0.01, η^2^ = 0.04).

For the large level of amount there was no significant main effect of social role (*F*[2,191] = 0.90, *p* < 0.01, η^2^ = 0.04, **Table 2**). However, the main effect of financial activity was observed (*F*[1.49,284.08] = 33.16, *p* < 0.001, η^2^ = 0.15, **Table 2**). There were significant differences between consumption and both saving (*p* < 0.001) and investing (*p* < 0.001) in money assigned. There was no significant difference between saving and investing. Moreover, the interaction between social role and financial activity was significant (*F*[2.98,284.08] = 7.67, *p* < 0.001, η^2^ = 0.07).

**FIGURE 1:**
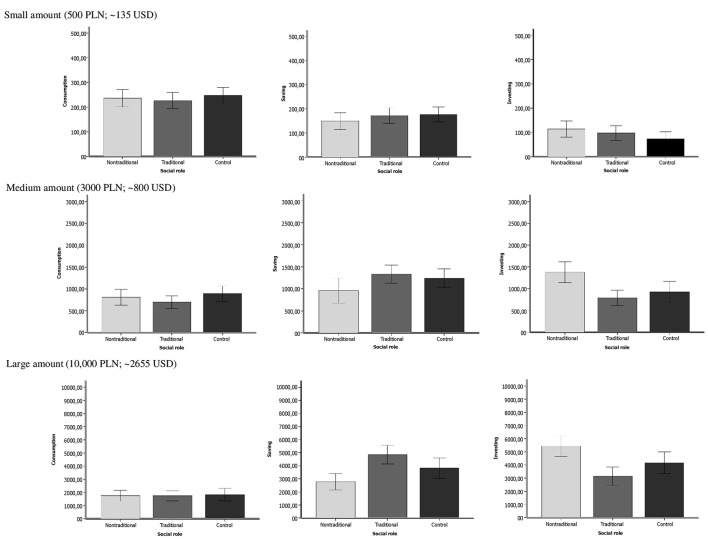
**Money assigned to consumption, saving and investing by traditional, nontraditional and control group for different levels of amount (Mean amount in PLN)**.

To correctly interpret the obtained interactions and to check the relationships presented above, the small, medium and large amount subsets were divided according to the three categories of financial activity (consumption, saving, and investing) and ANOVAs were run to compare each social role category (traditional, non-traditional, control). The average amount of money assigned to consumption, saving and investing for small, medium and large amount levels are presented in **Figure 1**. The results of the ANOVA showed that for all amount levels of money divided, the difference between the three analyzed groups (traditional, non-traditional and control) in the amount of money spent on consumption was not significant (small amount – *F*[2,192] = 0.38, *p* = 0.69, η^2^ = 0.004; medium amount – *F*[2,192] = 1.48, *p* = 0.23, η^2^ = 0.02; large amount *F*[2,192] = 0.61, *p* = 0.94, η^2^ = 0.001). Furthermore the analysis showed that there were no significant differences between the three social role conditions in saving when the amount of money for disposal was small (*F*[2,192] = 0.82, *p* = 0.44, η^2^ = 0.01). For the medium amount, ANOVA indicated that the difference between the social role groups was significant (*F*[2,192] = 2.77, *p* < 0.05, η^2^ = 0.04), with the non-traditional group’s propensity to save being lower than that of the traditional group (*t*[192] = 2.25, *p* < 0.05, *Cohen’s d* = 0.38). Furthermore, a significant manipulation effect was observed for the propensity for saving a large amount (*F*[2,192] = 8.502, *p* < 0.001, η^2^ = 0.08) Further *t*-tests showed that participants who were activated by the traditional social role of women tended to save more than those from the non-traditional (*t*[191] = 4.12, *p* < 0.001, *Cohen’s d* = 0.76) or control (*t*[191] = 2.04, *p* < 0.05, *Cohen’s d* = 0.34) groups. Furthermore, participants in the control group were prone to save more money than the non-traditional group (*t*[191] = 2.07, *p* < 0.05, *Cohen’s d* = 0.37). The last three ANOVAs were used to compare the means of the amount of money destined for investment in the three social role conditions. There was no significant difference observed when the divided amount was small (*F*[2,192] = 1.73, *p* = 0.18, η^2^ = 0.02). For the medium and large amount level, ANOVA indicated that the differences between the groups were significant (medium amount: *F*[2,192] = 7.68, *p* < 0.001, η^2^ = 0.07; large amount *F*[2,192] = 8.99, *p* < 0.001, η^2^ = 0.09). The contrast tests showed that participants from the non-traditional group were more prone to invest than those from the traditional or control groups if they were asked to divide the medium or large amount of money. Furthermore, participants from the non-traditional group who allocated medium and large amounts were more prone to invest than those from the traditional (medium amount: *t*[118] = 3.93, *p* < 0.001, *Cohen’s d* = 0.69; large amount: *t*[126] = 4.39, *p* < 0.001, *Cohen’s d* = 0.77) and control (medium amount: *t*[128] = 2.64, *p* < 0.01, *Cohen’s d* = 0.46; large amount: *t*[126] = 2.23, *p* < 0.05, *Cohen’s d* = 0.34) groups.

The results of the first study showed the influence of the activation of different social roles of women on the money spending pattern. Presenting women in the traditional social role (housewife) activated a stronger saving than investing tendency (financial behavior more typical for women). On the contrary, presenting women in the non-traditional social role (professional) activated a stronger investing than saving tendency (financial behavior more typical of men). This result was observed when the medium and large amounts of money were allocated. However, the level of money allocated for consumption stayed the same, independently from the activation of the social role (neutral, traditional and non-traditional). This result (or, in fact, lack of significant results) prompted the question of whether there is really no influence of activation of traditional vs. non-traditional social roles on consumption. Maybe there is no influence on the level of consumption (amount of money allocated to it) but on the quality of it (how much money is allocated for different goods).

## Study 2 – the Influence of Women’s Social Roles on their Consumer Decisions

The study presented here aimed to check whether, despite no differences in the level of consumption in the first study, any qualitative differences in consumer choices could be observed according to the activation of different social roles of women. It was hypothesized that that activation of different social roles of women would affect women’s consumer choices (Hypothesis 2).

### Method

#### Participants

A total of 196 women took part in the second study. In a similar way to Study 1, participants were students of the fourth and fifth years at University of Warsaw, aged 22 to 33 years (*M* = 24, *SD* = 1.89). The Ethics Board of the Faculty of Psychology at the University of Warsaw approved the study, which was carried out in accordance with the Board’s recommendations. At the end of the study, the participants were fully debriefed.

#### Experimental Design and Materials

The study was conducted with three conditions: two experimental conditions and one control group. Participants were randomly assigned to each condition, where thinking of the traditional, non-traditional or neutral social roles of women was activated. The social role that was activated was the between subjects IV. The first within subjects IV was the consumption category, measured on five levels: appearance, food and hygiene, equipment, relaxation, other. The second within subjects IV was the level of the amount that participants were asked to distribute, measured on three levels: small (PLN 500; ∼ USD 135), medium (PLN 3000; ∼USD 800) and large (PLN 10,000; ∼USD 2655). Women’s propensity for different consumer choices indicated by the money amount assigned by participants to different consumption categories was the DV.

The social role was activated exactly the same way as in Study 1.

Women’s propensity for different consumer choices was measured using the tool consisting of three questions, in which participants were ask to distribute the small (question1), medium (question 2) and large (question 3) amount between five categories of consumption: appearance, food and hygiene, equipment, relaxation, other.

#### Procedure

The procedure in Study 2 was almost the same to that used in Study 1, with only one difference. After the experimental manipulation, the propensity for different consumer choices (appearance, food and hygiene, equipment, relaxation, other) was measured.

### Results

A 3 (social role) × 3 (amount level) × 5 (consumption category) mixed-design analysis of variance was conducted. The main effect of social role was not significant (*F*[2,188] = 1.03, *p* = 0.36, η^2^ = 0.01, **Table 3**). A significant main effect of the consumption category was observed (*F*[2.98,559.40] = 91.31, *p* < 0.001, η^2^ = 0.33, **Table 3**). There were significant differences in the amount of money assigned between appearance and relaxation (*p* < 0.001), food and hygiene (*p* < 0.001), equipment (*p* = 0.06) and others (*p* < 0.001). The differences between relaxation and food and hygiene (*p* < 0.001) and equipment (*p* < 0.001) was also significant. Moreover, a significant differences between equipment and food and hygiene (*p* < 0.001), equipment and others (*p* < 0.001) and between others and food and hygiene (*p* < 0.001) occurred. The main effect of amount level was also significant (*F*[1.27,238.75] = 1476.28, *p* < 0.001, η^2^ = 0.89, **Table 3**). The differences between small and both medium (*p* < 0.001) and large (*p* < 0.001) money levels in the amount of money assigned were significant. Moreover the difference between medium and large amount levels was also significant (*p* < 0.001).

**Table 3:** Mean amounts of money (in PLN) assigned depending on social role, consumption category and amount level.

	*M*	95% Confidence Interval
Social role
Non-traditional	878.21	[819.70, 936.72]
Traditional	821.20	[762.69, 879.71]
Control	866.79	[807.82, 925.77]
Consumption category
Appearance	1079.18	[964.04, 1194.31]
Food and hygiene	256.97	[221.85, 292.09]
Equipment	843.63	[720.53, 966.73]
Relaxation	1741.35	[1590.33, 1892.32]
Other	355.88	[234.34, 477.43]
Amount level
Small	104.38	[98.34, 110.43]
Medium	580.76	[545.87, 615.65]
Large	1881.06	[1799.54, 1962.59]

The two–way interactions between social role and amount level (*F*[2.54,238.75] = 0.56, *p* = 0.62, η^2^ = 0.01) and between social role and the consumption category (*F*[5.95,559.40] = 1.71, *p* = 0.11, η^2^ = 0.02) were not significant. However, a significant interaction between the amount level and consumption categories occurred (*F*[3.44,646.04] = 58.82, *p* < 0.001, η^2^ = 0.24).

The three–way interaction between social role, amount level and consumption categories was also significant (*F*[6.87,646.04] = 2.23, *p* < 0.05, η^2^ = 0.04).

For further analysis, the data set was split according to the levels of the variables involved in the significant interaction between social role, consumption category and amount levels. In a first step, the data set was divided into the three amount level subsets. Three 3 (social role) × 5 (consumption category) mixed-design ANOVAs were conducted. For the small amount level, the main effect of social role was not observed (*F*[2,189] = 0.19, *p* = 0.83, η^2^ = 0.002, **Table 4**). A significant main effect of consumption category occurred (*F*[2.93,554.08] = 114.10, *p* < 0.001, η^2^ = 0.38, **Table 4**). The differences in amount of money allocated between appearance and relaxation (*p* < 0.001), food and hygiene (*p* < 0.001), equipment (*p* < 0.001), others (*p* < 0.001) were significant. Relaxation differed significantly from food and hygiene *(p* < 0.001), equipment (*p* < 0.001) and others (*p* < 0.001) in the amount of money allocated. Significant differences were also observed between food and hygiene and equipment (*p* < 0.001), food and hygiene and others *(p* < 0.001) and equipment and others (*p* < 0.001). Furthermore the one-way interaction between the social role and consumption categories was significant (*F*[5.86,554.08] = 2.03, *p* < 0.001, η^2^ = 0.02). For the medium amount level, the main effect of social role was not significant (*F*[2,189] = 0.88, *p* = 0.42, η^2^ = 0.01, **Table 4**). A significant main effect of the consumption category was observed (*F*[2,189] = 0.88, *p* = 0.42, η^2^ = 0.01, **Table 4**). The appearance category differed significantly from relaxation (*p* < 0.01), food and hygiene (*p* < 0.001), equipment (*p* < 0.001), others (*p* < 0.001). A significant difference occurred in the allocation of money between relaxation and food and hygiene (*p* < 0.001), equipment (*p* < 0.001) and others (*p* < 0.001). Significant differences were also observed between food and hygiene and equipment (*p* < 0.001), food and hygiene and others (*p* < 0.001) and equipment and others (*p* < 0.001). Moreover the one-way interaction between social role and the consumption category was significant (*F*[3.03,571.72] = 2.64, *p* < 0.05, η^2^ = 0.01). For the large amount level, the main effect of social role was not significant (*F*[2,189] = 0.46, *p* = 0.63, η^2^ = 0.01, **Table 4**). However, the main effect of the consumption category was significant (*F*[2.15,405.7] = 91.13, *p* < 0.001, η^2^ = 0.33, **Table 4**). There were significant differences observed in the money allocated between the appearance category and relaxation (*p* < 0.001), food and hygiene (*p* < 0.001), others (*p* < 0.001). Significant differences also occurred in the allocation of money between relaxation and food and hygiene (*p* < 0.001), equipment (*p* < 0.001) and others (*p* < 0.001). Significant differences were also observed between food and hygiene and equipment (*p* < 0.001), food and hygiene and others (*p* = 0.056) and equipment and others (*p* < 0.001). Furthermore, the one-way interaction between the social role and consumption categories was significant (*F*[5.93,557.02] = 2.17, *p* < 0.05, η^2^ = 0.03).

**Table 4:** Mean amounts of money (in PLN) assigned depending on social role and consumption category for small, medium and large amount level.

	Small amount *M*; 95% CI	Medium amount *M*; 95% CI	Large amount *M*; 95% CI
Social role
Non-traditional	106.59; [96.18, 117.01]	603.19; [543.08,663.30]	715.90; [635.14, 796.66]
Traditional	104.53; [94.12, 114.95]	548.70; [488.60, 608.81]	660.88; [580.12, 741.64]
Control	101.98; [91.57, 112.40]	590.55; [530.44, 650.65]	679.16; [598.40, 759.92]
Consumption category
Appearance	216.59; [196.29, 236.88]	860.00; [766.25, 953.75]	2157.90; [1871.90, 2443.91]
Food and hygiene	101.43; [87.86, 115.00]	228.52; [189.54, 267.49]	439.50; [365.35, 513.65]
Equipment	30.50; [17.58, 43.41]	494.79; [360.68, 628.91]	2002.82; [1696.40, 2309.24]
Relaxation	157.24; [139.83, 174.65]	1157.94; [1037.54, 1278.35]	3902.66; [3528.07, 4277.25]
Others	016.09; [6.52, 25.67]	162.81; [88.57, 237.05]	902.43; [594.73, 1210.14]

**FIGURE 2:**
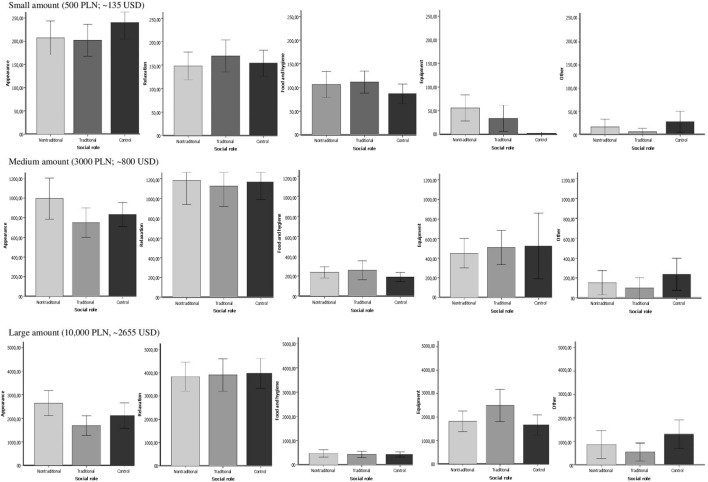
**Money assigned to consumption categories by each social role group for different amount levels (Mean amount in PLN)**.

In order to correctly interpret the results, the small, medium and large amount data subsets were divided according to the five categories of consumption (appearance, food and hygiene, equipment, relaxation, other) and one-way ANOVAs were run to compare each social role category (traditional, non-traditional, control). The descriptive statistics are presented in **Figure 2**. No significant differences were observed between the experimental groups in the amount spent on the appearance category of consumption when women were asked to divide the small amount of money (*F*[2,189] = 1.514, *p* = 0.22, η^2^ = 0.02). However, when medium and large amounts of money were distributed, significant differences between the experimental groups were observed (medium amount: *F*[2,188] = 6.96, *p* = 0.001, η^2^ = 0.07; large amount: *F*[2,189] = 6.34, *p* < 0.01, η^2^ = 0.06). Further *t*-tests showed that for the medium and large amount level the non-traditional group tended to spend more money on the consumption of appearance category than the traditional (medium: *t*[124] = 3.35, *p* = 0.001, *Cohen’s d* = 0.59); large: *t*[189] = 3.28, *p* = 0.001, *Cohen’s d* = 0.53) and control group (medium: *t*[115] = 2.65, *p* < 0.01, *Cohen’s d* = 0.47; large: *t*[189] = 2.84, *p* < 0.01, *Cohen’s d* = 0.50). An ANOVA test showed no difference between the experimental groups (activation of social roles) in terms of the sum of money assigned to food and hygiene purposes when the level of the divided amount was small (*F*[2,189] = 2.61, *p* = 0.08, η^2^ = 0.03) or medium (*F*[2,189] = 0.113, *p* = 0.89, η^2^ = 0.001). Significant differences were observed only when participants were asked to dispose of a large amount of money (*F*[2,189] = 6.09, *p* < 0.01, η^2^ = 0.06). Further analysis showed that the non-traditional group was prone to spend less money on food and hygiene consumption than other social role groups (non-traditional vs. traditional: *t*[135] = 3.49, *p* = 0.001, *Cohen’s d* = 0.6; non-traditional vs. control: *t*[110] = 2.30, *p* < 0.05, *Cohen’s d* = 0.41). Significant differences between the three social role groups in the amount of money assigned to equipment were observed when people were asked to divide the small amount of money (*F*[2,189] = 9.22, *p* < 0.001, η^2^ = 0.09). However, the ANOVA test did not indicate differences between the groups when distributing the medium (*F*[2,189] = 0.70, *p* = 0.50, η^2^ = 0.01) and large amount (*F*[2,189] = 2.20, *p* = 0.11, η^2^ = 0.02). Further *t*-tests showed that the non-traditional group was prone to spending more money on equipment than other groups (non-traditional vs. traditional: *t*[189] = 2.44, *p* < 0.05, *Cohen’s d* = 0.35; non-traditional vs. control: *t*[189] = 4.28, *p* < 0.001, *Cohen’s d* = 0.73) on the small level of the divided amount. An ANOVA test showed no difference between the groups in terms of the sum of money they assigned to relaxation, independently of the level of the divided amount (small: *F*[2,189] = 0.50, *p* = 0.61, η^2^ = 0.005, medium: *F*[2,189] = 2.45, *p* = 0.09, η^2^ = 0.03, large: *F*[2,189] = 0.42, *p* = 0.66, η^2^ = 0.004). Furthermore, no significant differences occurred between the three social role groups in their propensity to spend money on the other consumption category for the small (*F*[2,189] = 1.58, *p* = 0.21, η^2^ = 0.02), medium (*F*[2,189] = 1.11, *p* = 0.33, η^2^ = 0.01) and large (*F*[2,189] = 2.03, *p* = 0.14, η^2^ = 0.02) amount level.

The first study showed that the amount of money allocated to consumption (compared to financial goals like saving and investing) was constant in all conditions of social role activation. The second study, as was predicted, showed that depending on the activation of the social role (traditional or non-traditional) the goal of consumption was different. The clearest results were visible when the largest amount of money was allocated. The activation of the traditional social role of women increased the amount of money spent on food and hygiene products – thus more collectivistic and functional goals, where purchased products are used more for the whole family and not individually. Conversely, while the non-traditional social role of women was activated (closer to men’s role), more money was allocated for goals connected with appearance goods and equipment (e.g., home appliances, GSM phones, computer equipment) – thus more hedonistic and for individual usage.

## Study 3 – the Influence of Women’s Social Roles on their Judgments and Willingness to Buy an Advertised Product

The previous studies (Study 1 and Study 2) confirmed that the activation of the traditional vs. non-traditional social role of women could have an influence on the pattern of money allocation: (a) The proportion between money spent on saving, investing and current consumption; and (b) The preferred category of products and services for consumption. The goal of this study (Study 3) was to examine whether the activation of different social roles of women by a model appearing in advertising can affect the judgment of the advertised product (among others its price, as an indirect indicator of quality assessment), and willingness to buy an advertised product. It was predicted that activation of the traditional social role (model as housewife) would decrease product judgment, whereas the activation of the non-traditional social role (model as professional) would increase product judgment, its price and willingness to buy (Hypothesis 3).

### Method

#### Participants

Ninety women took part in the third study. They were students of the University of Warsaw. Participants were aged 19–48 years (*M* = 22, *SD* = 5.01). The Ethics Board of the Faculty of Psychology at the University of Warsaw approved the study, which was carried out in accordance with the Board’s recommendations. At the end of the study, the participants were fully debriefed.

#### Experimental Design and Materials

The study was conducted with three conditions: two experimental conditions and one control group. Participants were randomly assigned to each condition where thinking of the traditional, non-traditional or neutral social roles of women was activated. The social role activated was the between subjects IV. There were six DVs: assessed product price, quality, look, aroma, color and willingness to buy the product.

The social role was activated using an advertisement for tea. Three versions of the ad were created especially for the purpose of the experiment. Each version of the advertisement consisted of a photo of the same woman in a bright room, standing next to a table with chairs with a cup of tea in her hand (exactly the same photos as those used in Studies 1 and 2), a picture of a box of tea on the table and a slogan (“Perfect tea for you!”). The only difference between the three versions of the advertisement was the outfit worn by the model (the same as used in studies I and II). In the first version of the ad the model was in a traditional social role, in everyday clothes with a kitchen towel and oven glove. The second picture showed a woman in a non-traditional social role, wearing clothes suggesting her professional position. The third version of the ad was the control, showing a woman who was neutral in terms of social role, wearing casual clothes.

The assessment of the advertised product and willingness to buy the product was measured using the set of questions in which participants were asked: (a) To assess an expected price of the advertised product; (b) To rate the product on four dimensions: quality, look, aroma and color on a 5-point scale of -2 (very bad) to +2 (very good); and (c) To assess their willingness to buy the product on a 5-point scale of -2 (certainly not) to +2 (certainly yes).

#### Procedure

The procedure in Study 3 was almost the same as those used in Studies 1 and 2, but after the experimental manipulation (looking at one version of the advertisement), in Study 3 participants were asked to judge the advertised product (its price, quality, look, aroma and color) and willingness to buy the product.

### Results

MANOVA was used to examine differences in the judgment of the product across the social role categories. Results showed a significant effect of thinking of social role activation (Wilks’ λ = 0.67; *F*[12,160] = 3.01, *p <* 0.001, η^2^ = 0.18). Follow-up univariate ANOVA using Bonferroni corrections showed a significant main effect of social role on judgment of price of the product (*F*[2,85] = 8.81, *p <* 0.001, η^2^ = 0.17, **Table 5**). The non-traditional group assessed the price of the product higher than both traditional (*p* < 0.001) and control (*p* < 0.05) groups. There were no significant differences observed between the traditional and control group in their assessment of the product price. ANOVA also showed a significant main effect of social role on judgment of quality of the product (*F*[2,85] = 5.59, *p* < 0.01, η^2^ = 0.12, **Table 5**). Women in the non-traditional group rated the product quality higher than participants from the traditional (*p* = 0.001) and control (*p* = 0.06) groups. Participants from the traditional and control groups did not differ in their rating of the quality of the product. A significant main effect of social role on judgment of look of the product was also observed (*F*[2,85] = 5.84, *p* < 0.01, η^2^ = 0.12, **Table 5**). The non-traditional group assessed the product look higher than both the traditional (*p* = 0.058) and control (*p* = 0.001) groups. No difference was observed between the traditional and control groups in their judgment of product look. ANOVA results also showed a significant main effect of social role on judgment of color of the product (*F*[2,85] = 3.36, *p* < 0.05, η^2^ = 0.07, **Table 5**). Women in the non-traditional group rated the product color higher than participants from the traditional (*p* < 0.05) and control (*p* < 0.05) groups. Participants from the traditional and control group did not differ in their rating of the color of the product. There were no significant main effects of social role on the judgment of aroma (*F*[2,85] = 0.78, *p* = 0.46, η^2^ = 0.02, **Table 5**) and willingness to buy the product (*F*[2,85] = 1.12, *p* = 0.33, η^2^ = 0.03, **Table 5**) observed in the results of the ANOVAs.

**Table 5:** The product judgment depending on social role.

	Social role		
	Non-traditional *M*; 95% CI	Traditional *M*; 95% CI	Control *M*; 95% CI
Product price (in PLN)	12.48; [10.43, 14.53]	6.34; [4.22, 8.46]	8.69; [6.64, 10.73]
Product quality	0.37; [0.02, 0.71]	-0.46; [-0.82, -0.11]	-0.10; [-0.45, -0.25]
Product look	0.50; [0.12, 0.89]	-0.04; [-0.44, 0.36]	-0.43; [-0.82, -0.05]
Product color	0.57; [0.19, 0.94]	0.00; [-0.39, 0.39]	-0.07; [-0.44, 0.31]
Product aroma	0.13; [-0.18, 0.45]	-0.14; [-0.47, 0.19]	0.07; [-0.25, 0.38]
Willingness to buy the product	-0.67; [-1.11, -0.49]	-1.00; [-1.32, -0.68]	-0.80; [-1.11, -0.49]

The results of Study 3 show that the activation of thinking of the non-traditional social role of women affected the judgment of the advertised product. As was expected, the activation of the non-traditional social role of women increased the assessment of product price, quality, look and color. However, there was no effect observed in terms of the judgment of product aroma and willingness to buy the advertised product. Contrary to expectation, the activation of thinking of the non-traditional social role of women did not significantly affect the judgment of the advertised product. Moreover, the results of Study 3 showed that the product advertised by the woman in the non-traditional social role was judged higher on the dimensions of price, quality, look and color than the product advertised by the woman in the traditional social role.

### Discussion

The main goal of the three studies was to verify how the activation of different social roles (traditional or non-traditional) may be reflected in women’s financial and consumer choices. It was expected that exposure to the non-traditional social role would make women more prone to invest their money, while the activation of the traditional role would increase the participants’ tendency to save money in safe financial instruments. Moreover, we hypothesized that the activation of the non-traditional social role would increase women’s propensity to spend their money on the hedonistic products from the appearance category of consumption and services related to relaxation, while the activation of the traditional social role would increase the women’s tendency to spend money on functional consumption like food and hygiene products or equipment for the family. We also expected that the activation of the different social roles of women used in the advertisements may change women’s judgment of the advertised product and the willingness to buy it.

The results showed that activation of the non-traditional social role of woman increased the women’s tendency to invest and decreased their propensity to save money. The opposite results were obtained when the traditional social role was activated. Activating of the non-traditional social role of women (close to the traditional social role of a man) changed women’s choices in the way that brought them closer to the choices that are typical to men. According to earlier research (e.g., Bajtelsmit and Bernasek, 1996; Bajtelsmit and VanderHei, 1997; Seguino and Floro, 2003) men, due to their general risk tolerance, take more risky investment decisions than women and are less likely than women to save money. Based on our results, it can be presumed that the societal change that has taken place in recent decades and created a new social role for women (similar to the traditional social role of a man), has probably changed women’s investment and saving choices and made them closer to those of men.

In the presented study there was no effect of the activation of different social roles of women on the propensity to spend money on current consumption. However, the results showed that the activating of the non-traditional social role of women raises the propensity to spend money on products and services for individual use and reduces the willingness to buy goods that will be shared with other members of the household. Earlier research (Czuchaj-Ładód and Stopyra, 2015) indicated that in consumer activities men are more focused on their own needs while women exhibit rather communal behavior due to their higher tendency to take care for the household. In our study, the activation of the non-traditional social role of women resulted in the female respondents’ choices being closer to the choices that are typical for men (preference of products and services for individual use), therefore it can be presumed that the societal change that has taken place in recent decades, involving the emergence of a non-traditional role of women, may also affect women’s consumer choices.

Moreover, the study proved that the change of women’s social role may not only influence consumer choices, but may also affect the judgment of an advertised product when it is presented by woman in the non-traditional social role. Participants who watched the ad presenting the woman in the non-traditional social role estimated the product’s quality, look, color and its price higher than those exposed to the ad with a model in the traditional or neutral social role. Such results may suggest that products advertised by women in non-traditional social roles may be perceived as being more exclusive, and of better and higher quality than products advertised by women in traditional social roles. There were no differences observed in terms of women’s willingness to buy an advertised product. One of the possible explanations for this is that the product wasn’t attractive to the study participants. Therefore, it would be worth verifying whether the results observed would be the same if the advertised product was different, e.g., one that is more attractive for the consumers.

The participants in the first study were prone to spend more money on consumption than both on savings and investments when asked to apportion small amounts of money. However, when they assigned medium and large amounts, their propensity to save and invest was significantly greater than their tendency to consume. The results are in accordance with Keynes’ theory, showing that in the short term, the APC decreases with an increasing disposable income. Moreover, the results of the second study showed that when asked to imagine a small amount, participants were prone to spend more money on the appearance category of consumption than on relaxation, and allocated more money on food and hygiene products than on equipment. However, the effects were exactly the opposite for medium to large amounts. A small amount gave participants little opportunity to fulfill their relaxation and equipment goods needs because the cost of most of the products and services in those consumption categories was greater than PLN 500. At the same time, participants’ needs for appearance products and services, as well as for food and hygiene products, could easily be fulfilled (at least partially) with a small amount of money. When participants were asked to spend a medium or large amount, they had many more relaxation and equipment product alternatives; thus, their propensity to spend on those consumption categories was probably higher.

The presented studies provide an important starting point for scholars interested in understanding the financial choices and product judgments of women in contemporary societies where societal change is taking place. The results of the studies conducted give some evidence that the new, non-traditional social role of women that is often presented in the media may affect women’s everyday financial choices and product judgments. However, one of the possible limitations of the studies is that we did not measure the participants’ valuation of the traditional and non-traditional roles of women. In further studies it would be worth verifying the results taking into account how the different social roles are valued by the participants, because role congruity theory posits that alignment with valued social roles would elicit rather positive reactions whereas misalignment with valued social roles could elicit negative reactions (Eagly and Karau, 2002; Diekman and Eagly, 2008).

## Author Contributions

KS planned the research, designed the studies and analyzed the data. AT assisted with data analyses. KS, AT, and DM wrote the manuscript and provided inputs during revisions.

## Conflict of Interest Statement

The authors declare that the research was conducted in the absence of any commercial or financial relationships that could be construed as a potential conflict of interest.

The reviewer AJC and handling Editor declared their shared affiliation, and the handling Editor states that the process nevertheless met the standards of a fair and objective review.

## References

[B1] Arcimowicz K. (2003). *Obraz Mȩżczyzny w Polskich Mediach. Prawda, fałsz, Stereotyp*. Gdańsk: Gdańskie Wydawnictwo Psychologiczne.

[B2] Arcimowicz K. (2008). “Przemiany mȩskości w kulturze współczesnej,” in *Nowi Mȩżczyźni? Zmieniajkace sike Modele Mȩskości we Współczesnej Polsce* ed. Fuszara M. (Warszawa: Wydawnictwo Trio) 41–64.

[B3] Astin A. W. Oseguera L. Sax L. J. Korn W. S. (2002). *The American Freshman: Thirty-Five Year Trends*. Los Angeles: Higher Education Research Institute, UCLA.

[B4] Bajkowski T. (2010). *Kobiecość i Mȩskość w Percepcji Młodzieży Akademickiej*. Warszawa: Wydawnictwo Akademickie ŻAK.

[B5] Bajtelsmit V. L. Bernasek A. (1996). Why do women invest differently than men? *Financ. Counsel. Plan.* 7 1–10.

[B6] Bajtelsmit V. L. Bernasek A. Jianakoplos N. A. (1999). Gender differences in defined contribution pension decisions. *Financ. Serv. Rev.* 8 1–10. 10.1016/S1057-0810(99)00030-X

[B7] Bajtelsmit V. L. VanderHei J. A. (1997). “Risk aversion and retirement income adequacy,” in *Positioning Pensions for the Year 2000* ed. Mitchell O. S. (Philadelphia: University of Pennsylvania Press) 45–66.

[B8] Barke R. P. Jenkins-Smith H. Slovic P. (1997). Risk perceptions of men and women scientists. *Soc. Sci. Q.* 78 167–176.

[B9] Barska A. (2005). “Tożsamość społeczno-kulturowa płci w kontekście ponowoczesnego świata,” in *Tożsamość Społeczno-Kulturowa Płci* eds Barska A. Mandal E. (Opole: Uniwersytet Opolski) 15–23.

[B10] Bator J. (1998). *Wizerunek Kobiety w Reklamie Telewizyjnej.* Warszawa: Wydawnictwo Instytutu Spraw Publicznych.

[B11] Byrnes J. P. Miller D. C. Schafer W. D. (1999). Gender differences in risk taking: a meta-analysis. *Psychol. Bull.* 125 367–383. 10.1037/0033-2909.125.3.367

[B12] Courtney A. Lockeretz S. W. (1971). A woman’s place: an analysis of roles portrayed by women in magazine advertisements. *J. Market. Res.* 8 92–95. 10.2307/3149733

[B13] Czuchaj-Ładód K. Stopyra J. (2015). *Gender Factor, Mȩskie branże w Kobiecych Rȩkach*. Available at: http://genderfactor.eu/files/GenderFactor_MeskieBranzeWKobiecychRekach_Raport_Maj2015.pdf

[B14] Diekman A. B. Eagly A. H. (2000). Stereotypes as dynamic constructs: women and men of the past, present, and future. *Pers. Soc. Psychol. Bull.* 26 1171–1188. 10.1177/0146167200262001

[B15] Diekman A. B. Eagly A. H. (2008). “Of men, women, and motivation: a role congruity account,” in *Handbook of Motivation Science* eds Shah J. Y. Gardner W. L. (New York, NY: Guilford Press) 434–447.

[B16] Diekman A. B. Goodfriend W. (2006). Rolling with the changes: A role congruity perspective on gender norms. *Psychol. Women Q.* 30 369–383. 10.1111/j.1471-6402.2006.00312.x

[B17] Diekman A. B. Johnston A. M. Loescher A. L. (2013). Something old, something new: evidence of self-accommodation to gendered social change. *Sex Roles* 68 550–561. 10.1007/s11199-013-0263-6

[B18] Eagly A. H. Diekman A. B. (2003). “The malleability of sex differences in response to changing social roles,” in *A Psychology of Human Strengths* eds Aspinwall L. G. Staudinger U. M. (Washington, DC: American Psychological Association) 103–115.

[B19] Eagly A. H. Karau S. J. (2002). Role congruity theory of prejudice toward female leaders. *Psychol. Rev.* 109 573–598. 10.1037/0033-295X.109.3.573 12088246

[B20] Eagly A. H. Wood W. Diekman A. B. (2000). “Social role theory of sex differences and similarities: a current appraisal,” in *The Developmental Social Psychology of Gender* eds Eckes T. Trautner H. M. (Mahwah, NJ: Erlbaum) 123–174.

[B21] England P. (2006). “Toward gender equality: progress and bottlenecks,” in *The Declining Significance of Gender?* eds Blau F. D. Brinton M. C. Grusky D. B. (New York, NY: Russell Sage Foundation) 245–264.

[B22] Faff R. Mulino D. Chai D. (2008). On the linkage between financial risk tolerance and risk aversion. *J. Financ. Res.* 31 1–23. 10.1111/j.1475-6803.2008.00229.x

[B23] Fawcett Society. (2007). *Saving Lives: Women’s Lifetime Savings Patterns*. London: Fawcett Society.

[B24] Fullerton H. N. (1999). Labor force participation: 75 years of change, 1950–1998 and 1998–2025. *Month. Lab. Rev.* 122 3–12.

[B25] Grable J. (2000). Financial risk tolerance and additional factors that affect risk taking in everyday money matters. *J. Bus. Psychol.* 14 625–630. 10.1023/A:1022994314982

[B26] Hall R. E. Taylor J. B. (1993). *Macroeconomics* 4th Edn. New York, NY: W.W. Norton.

[B27] Hallahan T. A. Faff R. W. McKenzie M. D. (2004). An empirical investigation of personal financial risk tolerance. *Financ. Serv. Rev.* 13 57–78.

[B28] Hinz R. P. McCarthy D. D. Turner J. A. (1997). “Are women conservative investors?: gender differences in participant-directed pension investments,” in *Positioning Pensions for the Twenty-First Century* eds Gordon M. S. Mitchell O. S. Twinney M. M. (Philadelphia: University of Pennsylvania Press) 99–106.

[B29] Hungerford T. (1999). *Saving for a Rainy Day: Does Pre-retirement Access to Retirement Savings Increase Retirement Saving?* Washington, DC: Mimeo, Social Security Administration.

[B30] Keynes J. M. (1936/2014). *The General Theory of Employment, Interest, and Money*. London: Macmillan.

[B31] Mager J. Helgeson J. G. (2011). Fifty years of advertising images: Some changing perspectives on role portrayals along with enduring consistencies. *Sex Roles* 64 238–252. 10.1007/s11199-010-9782-6

[B32] Maison D. (2013). *Polak w Świecie Finansów*. Warszawa: PWN.

[B33] Maison D. Rudzińska J. (2009). Obraz kobiety i mȩżczyzny w polskiej reklamie. *Market. Rynek* 11 28–33.

[B34] Neelakantan U. (2010). Estimation and impact of gender differences in risk tolerance. *Economic Inquiry* 48 228–233. 10.1111/j.1465-7295.2009.00251.x

[B35] Samuelson P. A. Nordhaus W. D. (1998). *Economics* 6th Edn New York, NY: McGraw-Hill Companies.

[B36] Seguino S. Floro M. S. (2003). Does gender have any effect on aggregate saving? An empirical analysis. *Int. Rev. Appl. Econ.* 17 147–166. 10.1080/0269217032000064026

[B37] Snowdown B. Vane H. R. (2005). *Modern Macroeconomics: Its Origins, Development and Current State*. Cheltenham: Edward Elgar Publishing.

[B38] Williams J. E. Best D. L. (1990). *Measuring Sex Stereotypes: A Multination Study*. Newbury Park, CA: Sage Publication.

[B39] Williams J. E. Satterwhite R. C. Best D. L. (1999). Pancultural gender stereotypes revisited: the five factor model. *Sex Roles* 40 1–13. 10.1023/A:1018831928829

